# Supplementary material for: Tracing Worldwide Turkey Genetic Diversity Using D-loop Sequence Mitochondrial DNA Analysis
Source: Animals (Basel). 2019 Nov 1;9(11):897. doi: 10.3390/ani9110897 (PMC6912331; doi:10.3390/ani9110897)

Figure S1. Median-joining haplotype network. The relationships between haplotypes identified in domesticated *M. gallopavo*, including 293 individuals, commercial lines' and arqueological samples; and 31 haplotypes are showns.

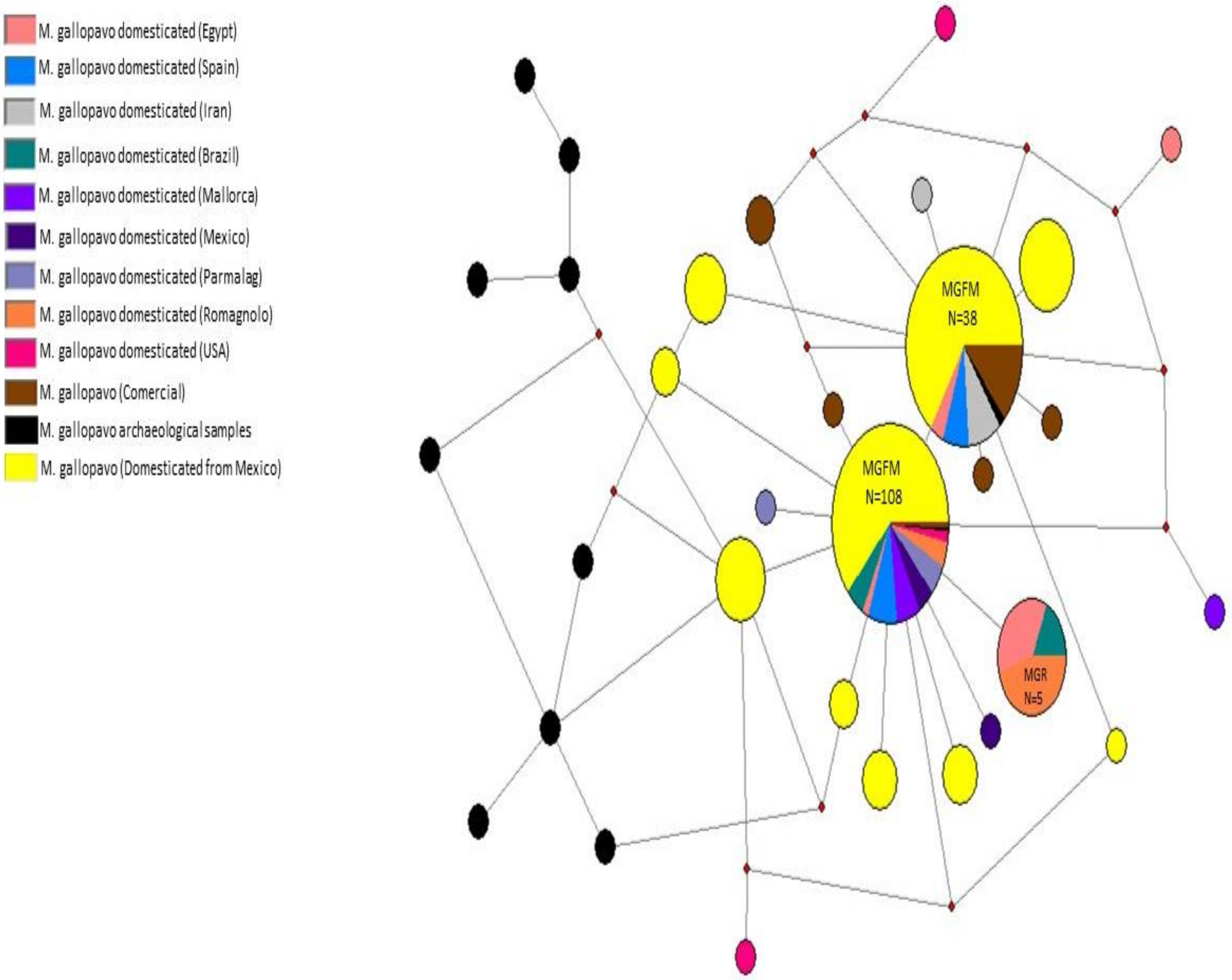

Supplement: Supplementary file 1 [file animals-09-00897-s001.zip › animals-591533-supplementary-proof done/Figure S1.pdf]
